# PREDICT version 3.1 overestimates survival in older women with breast cancer: a population-based external validation

**DOI:** 10.1016/j.breast.2026.104892

**Published:** 2026-08-01

**Authors:** Julia N. Wolbink, Leti van Bodegom-Vos, Luuk C.A. Ciere, Saskia le Cessie, Hein Putter, Anna Z. de Boer, Willeke van der Plas-Krijgsman, Frederiek van den Bos, Gerrit-Jan Liefers, Johanneke E.A. Portielje

**Affiliations:** a Department of Medical Oncology, Leiden University Medical Center, Albinusdreef 2, Leiden, 2333 ZA, the Netherlands; b Department of Biomedical Data Sciences, Leiden University Medical Centre, Albinusdreef 2, Leiden, 2333 ZA, the Netherlands; c Department of Gerontology and Geriatrics, Leiden University Medical Center, Albinusdreef 2, Leiden, 2333 ZA, the Netherlands; d Department of Clinical Epidemiology, Leiden University Medical Centre, Albinusdreef 2, Leiden, 2333 ZA, the Netherlands; e Department of Oncology, Medical Center Haaglanden, P.O. Box 432, The Hague, 2501 CK, the Netherlands; f Department of Surgery, Leiden University Medical Center, Albinusdreef 2, Leiden, 2333 ZA, the Netherlands

**Keywords:** Breast cancer, Prediction, Geriatric oncology, PREDICT, Older patients, Comorbidity

## Abstract

**Background:**

Prediction models can guide breast cancer treatment decisions by estimating individualized prognosis and treatment benefit. Since older women with breast cancer are more heterogeneous and face more competing non–breast cancer mortality, model performance may differ from that observed in the general breast cancer population. We externally validated PREDICT version 3.1 in a population-based cohort of Dutch older women with breast cancer.

**Methods:**

Women aged ≥70 years with breast cancer were included from the Dutch Cancer Registry. Observed 5- and 10-year overall survival (OS) were estimated using Kaplan–Meier methods and compared with predicted outcomes. Calibration was assessed using smooth calibration curves and observed/expected (O/E) ratios. Discrimination was evaluated with area under the curve (AUC). Performance was examined across age groups and comorbidity strata.

**Results:**

We included 11.041 women (median age 75.8 years, IQR 72.8-81.6). PREDICT v3.1 overestimated 5-year OS by 7.2% (95% CI = 6.3-8.0) and 10-year OS by 12.1% (95% CI = 11.1-13.1). Calibration plots showed deviation from the ideal line and the O/E ratio was 1.39 (95% CI = 1.34-1.44) for 5-year and 1.30 (95% CI = 1.27-1.34) for 10-year. The AUC for 5- and 10-year OS was 0.75 (95% CI = 0.74-0.76) and 0.76 (95% CI = 0.75-0.77), respectively. Overestimation of survival increased with age and number of comorbidities.

**Conclusion:**

PREDICT v3.1 overestimates survival in older women, particularly those with comorbidities and increased age. Despite moderate discrimination, poor calibration limits clinical applicability in this population.

## Highlights

• PREDICT v3.1 overestimates overall survival in older patients with breast cancer.
• Moderate discrimination and poor calibration limits clinical applicability.
• Lack of comorbidity input limits prediction of competing mortality.

## Graphical abstract

**Figure:**
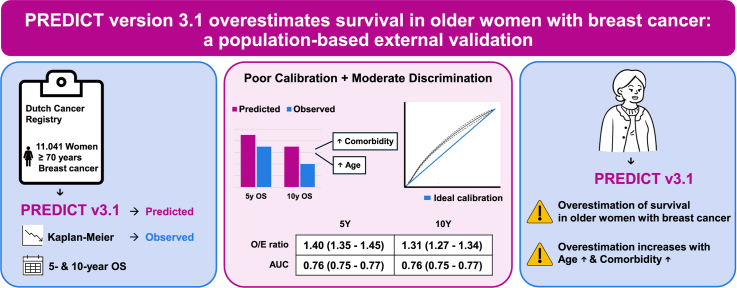

## 1 Introduction

Prediction models are becoming increasingly important in clinical decision-making, particularly in breast cancer care. By combining tumor and patient characteristics, these models provide individualized estimates of prognosis and treatment benefit. This enables patients and clinicians to make better-informed treatment decisions. Both Dutch and international breast cancer guidelines currently recommend use of the PREDICT breast prognostication and treatment benefit prediction tool to guide treatment decisions [1].

Prediction models used in clinical practice must be regularly updated and revalidated to maintain accuracy as healthcare practices and patient characteristics evolve. Model performance may vary across patient subgroups, making evaluation in the specific population in which it will be applied essential. This is particularly important for older patients, who represent a heterogeneous population with substantial variation in functional status, comorbidity burden, and life expectancy. They also face higher risks of competing non-cancer mortality [2,3]. Consequently, models developed in broader breast cancer populations may be less reliable in this age group.

PREDICT is regularly updated to enhance its predictive precision. The most recent publicly available version is 3.1 [4]. In this update, the baseline hazard introduced in version 3.0, which was derived from Eastern Cancer Registry data and reflected outcomes averaged across treated patients, was recalibrated to account for adjuvant treatment received. This recalibration increases estimated breast cancer mortality and consequently results in larger predicted absolute treatment benefits compared with version 3.0 [4]. However, evidence regarding what this update means for the model's performance in specific patient subgroups remains limited.

External validations of PREDICT v3.1 have been performed in the United Kingdom [5] and in the Netherlands [6]. Although overall performance in the general breast cancer population was acceptable, accuracy was reduced among women aged 75 years and older. The reasons for this diminished performance have not been systematically explored and the potential influence of comorbidity on model accuracy remains unclear.

Therefore, this study evaluates the validity of PREDICT v3.1 by examining its 5- and 10-year overall survival (OS) predictions in a large, population-based cohort of Dutch older women with breast cancer. We specifically assess performance across smaller age groups and comorbidity strata. This evaluation aims to determine the model's appropriateness for use in older women and to identify opportunities to improve its performance in this population.

## 2 Methods

### 2.1 Study population

For this validation study we used data from the Dutch Cancer Registry, including patients aged 70 years or older who were diagnosed with breast cancer in the Netherlands between 2005 and 2009 [7]. Eligible participants were female patients with primary breast cancer (stages I-III) who underwent surgery as the primary treatment.

Patients were excluded if data were missing for any predictor variables required by PREDICT v3.1: tumor size, number of positive lymph nodes, histological grade, or estrogen receptor status. This approach reflects the intended application of the model and avoids additional assumptions through imputation.

### 2.2 Handling of unavailable predictor variables

Several predictors required by PREDICT v3.1 were unavailable in the Dutch Cancer Registry. For these variables, assumptions were made for the primary analyses. To evaluate the impact of these assumptions, prespecified sensitivity analyses were performed (Appendix).

KI67 data was unavailable for all patients and was therefore coded as unknown, consistent with a previous validation study of the PREDICT v3.0 of Yi-Wen Hsiao et al. [8].

Smoking status was unavailable in the validation cohort. According to national data, the overall smoking prevalence in the Dutch population is 18.9%, and 15.2% among women [9]. Among individuals aged 65–74 years, the smoking prevalence decreases to 12.2%, and further to 6.5% among those aged 75 and older [9]. Given that all patients in the cohort were female, and the median age was above 75 years, it is reasonable to assume that only a small proportion of patients were smokers (around 3–6%). Since this proportion is low, all women were coded as ‘non-smokers’ in the primary analysis. However, as smoking is a key variable in the model, sensitivity analyses were performed by setting all individuals to 'smoker'.

Information on the route of detection was also unavailable. As national breast cancer screening in the Netherlands ends at age 75 and the cohort's median age exceeded this threshold, ‘detection through symptoms’ was used as the default setting in the primary analysis. A sensitivity analysis was performed by setting all women to ‘detection through screening’.

Lastly, the specific chemotherapy regimens administered were unknown. In the primary analysis, all women who received chemotherapy were assumed to have received standard-dose, anthracycline-based chemotherapy, reflecting regimens commonly administered to older women in that time period. A sensitivity analysis assuming ‘high dose, anthracycline- or taxane-based chemotherapy’ was also performed.

### 2.3 Predicted and observed outcomes

Predicted 5- and 10-year OS estimates from PREDICT v3.1 were calculated using a custom script in R version 4.4.0. based on the code of the developers of PREDICT v3.1 published on GitHub [10]. The output produced by the R script was validated by cross-checking results against the online PREDICT tool using a small set of test cases on 19 September 2025.

Observed 5- and 10-year survival rates were estimated using the Kaplan-Meier method for the entire cohort and predefined subgroups. Standard errors of the Kaplan-Meier estimates were calculated using Greenwood's formula.

### 2.4 Model performance

Model performance was evaluated by assessing calibration and discrimination.

Calibration was first assessed by comparing the mean predicted OS with observed Kaplan-Meier survival probabilities at 5 and 10 years. Absolute calibration differences were calculated as the difference between the mean predicted and observed survival probabilities (predicted - observed). 95% confidence intervals were calculated using Wald-type intervals based on the standard errors of the observed Kaplan-Meier estimates.

Subsequently, calibration was assessed graphically using smooth calibration curves and observed/expected (O/E) ratios following STRATOS recommendations [11]. Smooth calibration curves were generated using Cox models with the PREDICT linear predictor as only covariate, which provided estimates of observed mortality corresponding to each predicted risk level. Perfect calibration corresponds to an O/E ratio of 1.

Discrimination was evaluated by determining the area under the curve (AUC) of receiver-operator curves for 5- and 10-year OS. Risk regression was used to estimate survival probabilities while accounting for censoring.

### 2.5 Exploratory analyses

Additional exploratory analyses were performed to investigate the contribution of age and comorbidity to model calibration (Appendix)

First, subgroup calibration analyses according to tumor size, number of positive lymph nodes, histological grade, estrogen receptor status, and comorbidity burden were repeated separately for women <75 years and ≥75 years.

Second, to explore whether age-related differences in calibration persisted independently of comorbidity burden, analyses were restricted to women without recorded comorbidities, and calibration differences were assessed according to age group.

### 2.6 Statistical software

All statistical analyses were performed in R version 4.4.0 (The R Foundation, Vienna, Austria).

## 3 Results

### 3.1 Patient characteristics

**Fig. 1:**
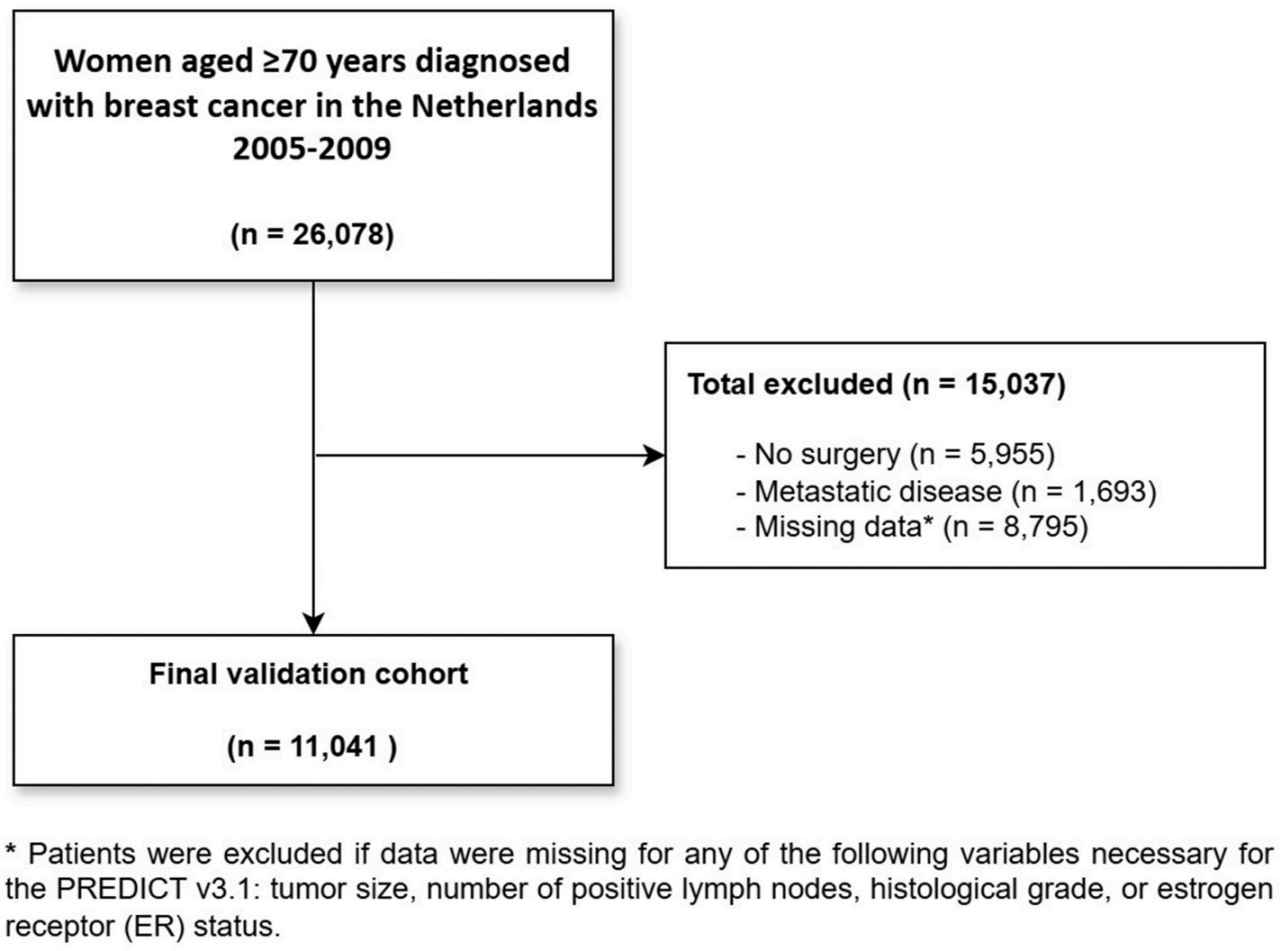
Flowchart illustrating patient selection for the study.

In total, 11.041 women from the Dutch Cancer Registry met the inclusion criteria and were included in the analysis (Fig. 1). Baseline characteristics are presented in Table 1. The median age was 75.8 years (IQR 72.8-81.6). Information on comorbidity was missing for approximately half of the cohort. Among 5508 women with available comorbidity data, 71.0% had one or more comorbidities. A total of 49.4% of women received endocrine therapy and 50.4% radiotherapy. Only 2.1% of the cohort received chemotherapy and 0.6% received trastuzumab, both are low but consistent with Dutch treatment trends in older women.

**Table 1:** Baseline characteristics and observed versus expected 5- and 10-year overall survival.

	N(%)	5 year			10 year		
		Predicted	Observed (s.e.)	Difference (95% CI)	Predicted	Observed (s.e.)	Difference (95% CI)
**Total**	11041 (100%)	81.6	74.4 (0.4)	7.2 (6.3 to 8)	60.3	48.2 (0.5)	12.1 (11.1 to 13.1)
**Age**							
<75	4899 (44.4%)	89.5	86 (0.5)	3.5 (2.5 to 4.4)	74.8	67.1 (0.7)	7.7 (6.3 to 9.1)
75-79	2629 (23.8%)	81.8	74.5 (0.9)	7.3 (5.6 to 8.9)	60.3	47 (1)	13.3 (11.3 to 15.3)
80-84	2322 (21%)	74.3	64 (1)	10.4 (8.4 to 12.3)	45.6	28 (1)	17.6 (15.7 to 19.6)
≥85	1191 (10.8%)	62.6	46.7 (1.4)	15.9 (13 to 18.7)	28.8	12.3 (1)	16.5 (14.5 to 18.6)
**N of comorbidities**							
0	1596 (14.5%)	83.7	82.8 (0.9)	0.9 (−1 to 2.7)	63.9	61.8 (1.3)	2.1 (−0.4 to 4.7)
1	1730 (15.7%)	82.1	75.4 (1)	6.6 (4.6 to 8.7)	60.6	48.8 (1.3)	11.8 (9.2 to 14.5)
2	1261 (11.4%)	81	72.3 (1.3)	8.7 (6.2 to 11.2)	59.4	45.2 (1.6)	14.2 (11.1 to 17.2)
3	620 (5.6%)	80.3	66.3 (1.9)	14 (10.3 to 17.7)	57.8	34.2 (2.2)	23.6 (19.3 to 27.9)
4	204 (1.8%)	78.5	56.4 (3.5)	22.1 (15.3 to 28.9)	53.7	28.8 (3.7)	24.9 (17.7 to 32.1)
≥5	97 (0.9%)	77.5	43.3 (5)	34.2 (24.4 to 44.1)	54.2	12.7 (5.9)	41.5 (29.9 to 53.1)
Unkown	5533 (50.1%)	81.3	74.3 (0.6)	7 (5.8 to 8.1)	59.9	47.6 (0.7)	12.3 (11 to 13.7)
**Tumor size (mm)**							
1-10	1734 (15.7%)	91.1	89.4 (0.7)	1.7 (0.2 to 3.1)	75.9	69.8 (1.2)	6.1 (3.8 to 8.4)
11-20	4406 (39.9%)	86.3	80.8 (0.6)	5.5 (4.3 to 6.7)	66.8	55.1 (0.8)	11.7 (10.1 to 13.2)
21-30	3115 (28.2%)	78	68.3 (0.8)	9.7 (8 to 11.3)	53.5	38.7 (0.9)	14.8 (13 to 16.5)
>30	1786 (16.2%)	66.9	54.7 (1.2)	12.2 (9.9 to 14.5)	40.8	26.7 (1.1)	14.1 (11.9 to 16.3)
**Tumor grade**							
1	2776 (25.1%)	88.6	82.3 (0.7)	6.3 (4.9 to 7.8)	70	56.7 (1)	13.3 (11.4 to 15.2)
2	5324 (48.2%)	84.1	77.5 (0.6)	6.6 (5.5 to 7.7)	62.4	50.1 (0.7)	12.3 (10.9 to 13.7)
3	2941 (26.6%)	70.4	61.5 (0.9)	8.9 (7.2 to 10.7)	47.2	36.7 (0.9)	10.5 (8.7 to 12.3)
**Positive nodes (N)**							
0	7263 (65.8%)	85.8	80.1 (0.5)	5.7 (4.8 to 6.6)	66	54.7 (0.6)	11.3 (10.1 to 12.6)
1-3	2636 (23.9%)	79.4	69.6 (0.9)	9.8 (8 to 11.5)	55.8	41.2 (1)	14.7 (12.7 to 16.6)
≥4	1142 (10.3%)	59.6	49.4 (1.5)	10.2 (7.3 to 13.1)	33.8	23 (1.3)	10.8 (8.2 to 13.4)
**Estrogen receptor status**							
Negative	1647 (14.9%)	63.6	57.3 (1.2)	6.3 (3.9 to 8.7)	44.7	35.1 (1.2)	9.5 (7.1 to 11.9)
Positive	9394 (85.1%)	84.7	77.4 (0.4)	7.3 (6.5 to 8.2)	63	50.5 (0.5)	12.5 (11.5 to 13.6)
**Progesterone receptor status**							
Negative	3611 (32.7%)	73.7	66.5 (0.8)	7.2 (5.7 to 8.8)	52.4	41.4 (0.9)	11 (9.3 to 12.7)
Positive	7430 (67.3%)	85.4	78.3 (0.5)	7.1 (6.2 to 8.1)	64.1	51.5 (0.6)	12.6 (11.4 to 13.8)
**HER2 receptor status**							
Negative	9936 (90%)	83	75.5 (0.4)	7.5 (6.6 to 8.3)	61.6	49.1 (0.5)	12.5 (11.5 to 13.5)
Positive	1105 (10%)	69.1	64.8 (1.4)	4.3 (1.5 to 7.1)	47.8	39.6 (1.5)	8.3 (5.3 to 11.3)
**Adjuvant endocrine therapy**							
No	5584 (50.6%)	81.4	75.4 (0.6)	6 (4.8 to 7.1)	62.6	52.6 (0.7)	10 (8.7 to 11.4)
Yes	5457 (49.4%)	81.8	73.4 (0.6)	8.4 (7.2 to 9.6)	57.9	43.5 (0.7)	14.3 (12.9 to 15.8)
**Adjuvant chemotherapy**							
No	10813 (97.9%)	81.8	74.5 (0.4)	7.3 (6.4 to 8.1)	60.4	48.1 (0.5)	12.3 (11.3 to 13.3)
Yes	228 (2.1%)	71.7	69.7 (3)	2.1 (−3.9 to 8)	52.4	50.5 (3.7)	1.9 (−5.3 to 9.2)
**Adjuvant radiotherapy**							
No	5477 (49.6%)	79.2	67.9 (0.6)	11.2 (10 to 12.5)	55.4	38.6 (0.7)	16.8 (15.5 to 18.2)
Yes	5564 (50.4%)	83.9	80.8 (0.5)	3.1 (2.1 to 4.2)	65	57.6 (0.7)	7.5 (6.1 to 8.8)
**Adjuvant trastuzumab**							
No	10975 (99.4%)	81.6	74.4 (0.4)	7.2 (6.4 to 8.1)	60.3	48.1 (0.5)	12.2 (11.2 to 13.2)
Yes	66 (0.6%)	68.6	77.3 (5.2)	−8.7 (−18.8 to 1.4)	50.3	57 (6.7)	−6.7 (−19.8 to 6.5)

### 3.2 Model performance

PREDICT v3.1 systematically overestimated overall survival at both 5 and 10 years.

The observed 5-year OS was 74.4% (s.e. 0.4%). The mean predicted 5-year OS was 81.6%, resulting in an overestimation of 7.2% (95% CI = 6.3-8.0) (Table 1). The discrepancy between predicted and observed 5-year OS increased with age. In the oldest age group, the mean overestimation reached 15.9% (95% CI = 13.0-18.7). Comorbidity also significantly affected prediction accuracy. Predictions for women without comorbidities were relatively accurate (mean difference of 0.9% with 95% CI = −1.0-2.7), whereas for women with five or more comorbidities, observed OS was nearly half the predicted value (mean difference of 34.2% with 95% CI = 24.4-44.1).

Overestimation was more pronounced for 10-year OS than for 5-year OS. The observed 10-year OS was 48.2% (s.e. 0.5%), while predicted OS was 60.3%, corresponding to an overestimation of 12.1% (95% CI = 11.1-13.1) (Table 1). Similar to 5-year OS, the prediction error for 10-year OS increased with both age and comorbidity.

**Fig. 2:**
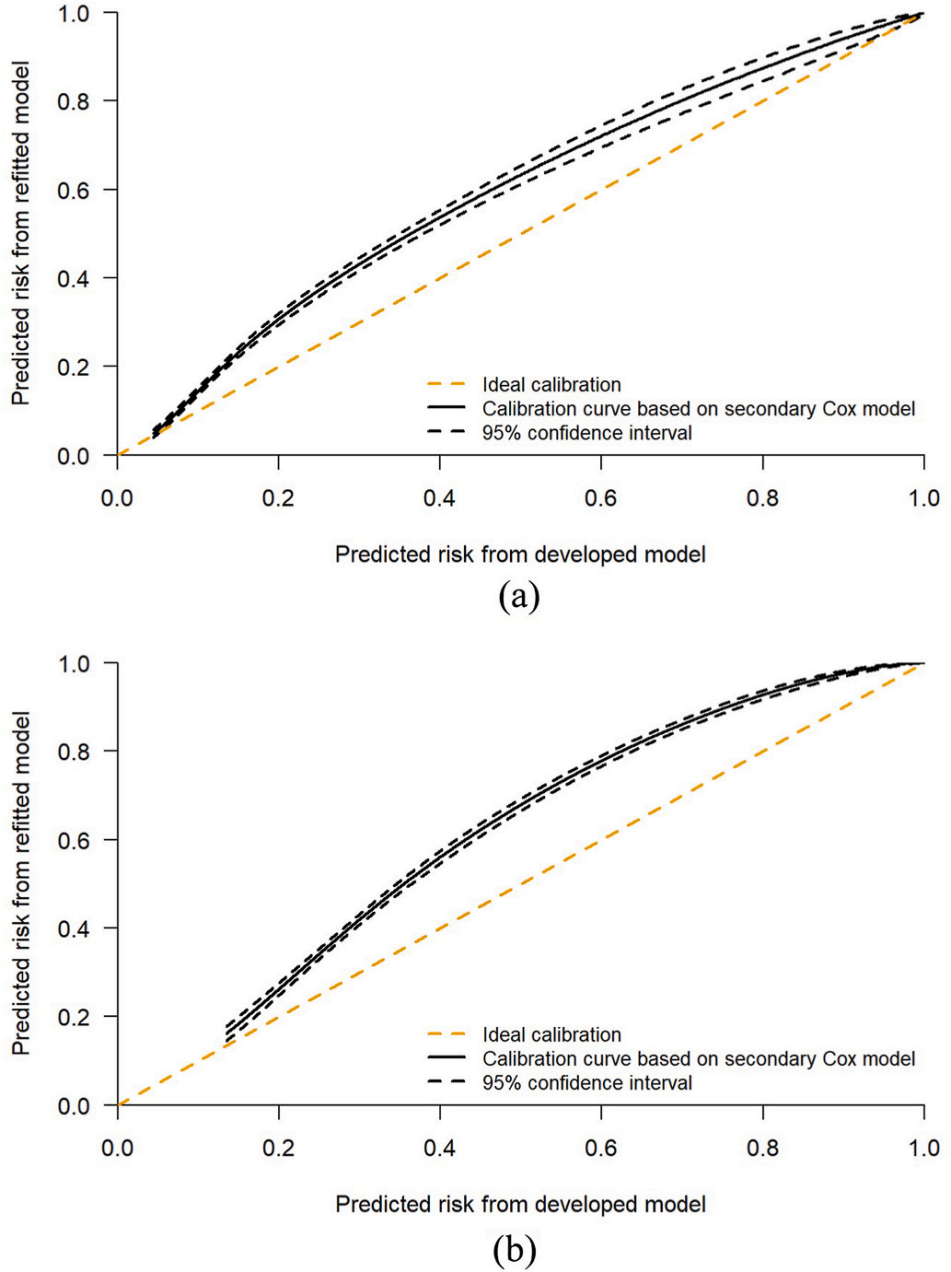
Calibration plots for 5-year (A) and 10-year (B) overall mortality.

Calibration of PREDICT v3.1 was subsequently assessed by using calibration plots for 5- and 10-year mortality (Fig. 2). Visual inspection showed poor calibration for both time horizons, with predicted outcomes deviating from the ideal line (x = y). The O/E ratio was 1.39 (95% CI = 1.34-1.44) for 5-year mortality and 1.30 (95% CI = 1.27-1.34) for 10-year mortality, indicating systematic overestimation of survival.

Finally, discrimination of PREDICT v3.1 was evaluated. The AUC was 0.75 (95% CI = 0.74-0.76) for 5-year OS and 0.76 (95% CI = 0.75-0.77) for 10-year OS. These AUC values indicate the model shows moderate discriminative ability. This indicates that the model generally assigns higher predicted survival to women who survived and lower predicted survival to those who did not.

A series of sensitivity analyses was performed (Appendix, Table A.1). Under the assumption that all women were smokers, PREDICT v3.1 continued to overpredict survival, with mean differences of 5.1% for 5-year OS (95% CI = 4.3-5.9) and 7.9% for 10-year OS (95% CI = 6.9-8.8). Assuming that all tumors were detected through screening rather than symptoms resulted in a greater overestimation, with mean differences of 8.8% for 5-year OS (95% CI = 8.0-9.6) and 14.4% for 10-year OS (95% CI = 13.4-15.3). Similarly, assuming treatment with high-dose anthracycline- or taxane-based chemotherapy instead of standard-dose anthracycline-based regimens resulted in an overestimation of 7.2% for 5-year OS (95% CI = 6.4-8.0) and 12.1% for 10-year OS (95% CI = 11.2-13.1).

### 3.3 Exploratory analyses

Additional exploratory analyses examined the contribution of age and comorbidity to calibration differences. When subgroup analyses were stratified by age (<75 versus ≥75 years), differences between predicted and observed survival were generally larger among women aged ≥75 years across tumor characteristics and comorbidity categories (Appendix, Table A.2). When analyses were restricted to women without recorded comorbidities, calibration differences were substantially reduced across age groups compared with the overall cohort. Moreover, the progressive increase in survival overestimation observed with increasing age was markedly reduced (Appendix, Table A.3).

## 4 Discussion

In this external validation study, PREDICT v3.1 tool overestimated both 5- and 10-year OS in Dutch older women with breast cancer. Although the model demonstrated moderate discrimination, calibration was poor. This indicates that while the model is able to rank women according to relative risk, its absolute survival estimates are overly optimistic in this population. Importantly, the discrepancy between predicted and observed survival increased progressively with age and each additional comorbidity, indicating a systematic pattern of miscalibration rather than random error.

The poorer calibration observed in older women is likely driven by the increasing impact of competing non-breast cancer mortality with advancing age. Whereas mortality in younger patients with breast cancer is primarily attributable by breast cancer–specific causes, older patients experience a shift toward non–breast cancer causes of death [2,3]. Because comorbidity is strongly associated with other-cause mortality [12,13], the absence of comorbidity in the model likely limits the model's ability to capture heterogeneity in competing mortality risks. The progressively worsening calibration across comorbidity strata supports this interpretation. Notably, predicted mortality was relatively accurate among women without comorbidities, suggesting that the model adequately captures breast cancer–specific mortality risk. Moreover, the progressive increase in survival overestimation observed with increasing age was markedly reduced when analyses were restricted to women without recorded comorbidities. Taken together, these findings suggest that incorporating comorbidity into the model may represent a promising strategy to improve estimation of competing mortality and reduce survival overestimation in older women.

Our findings should be interpreted in the context of previous external validations of PREDICT v3.1, which reported acceptable performance in the general breast cancer population but reduced accuracy among women aged 75 years and older [5,6]. In contrast to these studies, the present study demonstrates substantially poorer calibration in older women and shows that miscalibration worsens systematically with advancing age and increasing comorbidity. Earlier studies, however, did not explore potential mechanisms underlying this diminished performance. By evaluating narrower age strata and systematically assessing the impact of comorbidity, the present study provides insight into which subgroups are most affected and the likely mechanism underlying the reduced model performance in older women. Unlike previous validation studies, the present study not only demonstrates that model calibration deteriorates with advancing age, but also suggest that increasing comorbidity and the resulting competing mortality are important contributors to this deterioration.

Comparison with alternative prognostic tools externally validated in populations of older women with breast cancer further contextualizes these findings. Although direct comparisons should be interpreted cautiously because of differences in study populations and validation cohorts. External validation of PREDICT v2.0 showed good calibration for 5-year OS, with an standardized mortality ratio (SMR) of 1.07 (95% CI = 0.98–1.16), and only modest overestimation of 10-year OS, with an SMR of 1.12 (95% CI = 1.05–1.20). Discrimination of version 2.0 was moderate with a c-index of 0.73 (95% CI 0.70–0.75) [14]. Similarly, the PORTRET tool demonstrated near-ideal calibration for 5-year mortality in older women, with a SMR of 0.96 (95% CI = 0.93–0.99) and moderate discrimination with an AUC of 0.76 (95% CI = 0.75–0.76) [7]. Although PREDICT v3.1 offers slightly improved discrimination compared with PREDICT v2.0 and comparable discrimination to the PORTRET tool, its poorer calibration reduces its reliability for clinical decision-making in older women.

Given how PREDICT is used in clinical practice, calibration rather than discrimination is the key determinant of clinical usefulness, especially in older patients. In this population, which is particularly vulnerable to treatment-related adverse effects, clinical decision-making requires careful balancing of potential benefit against toxicity and impact on quality of life [15,16]. Overestimation of survival may directly translate into overestimation of treatment benefit, thereby increasing the risk of overtreatment and unnecessary exposure of older patients to treatment-related morbidity.

This study has several strengths. The large, population-based cohort of older women allowed for detailed analyses across narrow age groups and evaluation of the impact of comorbidity on model performance. In addition, the validation followed contemporary methodological standards set by STRATOS for assessment of discrimination and calibration [11] and incorporated multiple sensitivity analyses to account for unavailable predictor information.

Several limitations should be acknowledged. The validation cohort included women diagnosed between 2005 and 2009. However, this is unlikely to explain the observed miscalibration. Previous research has shown that relative survival among Dutch women with breast cancer aged 75 years and older has remained stable over the past two decades [17]. In addition, gains in life expectancy in this age group have been modest, with the expected remaining life expectancy of a 76-year-old Dutch woman increasing only slightly from 11.74 years in 2005–2009 to 12.15 years in 2022 [18]. Given this stability in both breast cancer-specific and all-cause survival, we consider this cohort to provide a valid and representative basis for evaluating model performance in contemporary older Dutch women.

Furthermore, several variables required for PREDICT v3.1 were unavailable in the validation cohort, including smoking status. Nevertheless, sensitivity analyses showed that even under the conservative assumption, such as classifying all women as smokers, PREDICT v3.1 still substantially overestimated survival (Appendix). Additional sensitivity analyses demonstrated that assuming screen-detected disease rather than symptomatic presentation, or high-dose anthracycline- or taxane-based chemotherapy instead of standard-dose anthracycline-based regimens, resulted in even greater overestimation of survival (Appendix). These findings suggest that missing predictor information is unlikely to explain the magnitude or direction of the observed miscalibration.

A further limitation is that comorbidity data were available for only approximately half of the cohort. However, mean predicted and observed survival probabilities among patients with available comorbidity data were comparable to those in the overall cohort, suggesting that this subgroup was broadly similar to the full cohort. Nevertheless, residual selection bias cannot be excluded, and the observed association between comorbidity and calibration should therefore be interpreted with caution.

Finally, although newer versions of PREDICT (v4.0 [5] and v4.1.1 [19]) have been developed, they were not evaluated in this study, as these versions are not yet publicly available. Moreover, neither version incorporates comorbidity or other explicit adjustments to improve competing mortality risk prediction. Given that underestimation of competing mortality appears to be the main source of miscalibration in older women, it seems unlikely that these updates would fully resolve the limitations observed here.

## 5 Conclusion

In conclusion, PREDICT v3.1 systematically overestimates survival in older women with breast cancer, particularly in those with comorbidities. Although the model demonstrates moderate discrimination, its poor calibration significantly limits its clinical applicability in this population. The model should therefore be used with caution when guiding treatment decisions in older women, and alternative tools with better calibration may be preferable. More broadly, our findings underscore the importance of independent external validation in target populations prior to clinical implementation and highlight the need for future prognostic models to better account for heterogeneity in competing mortality among older adults through incorporation of comorbidity.

## Data availability statement

Data from the Netherlands Cancer Registry (NCR) was used. This data is owned by the Netherlands Comprehensive Cancer Organisation (IKNL) and is available upon reasonable request.

## Ethical approval

Ethical approval was obtained from the Scientific Committee of Clinical Oncology, Leiden University Medical Center (LUMC), Leiden/Den Haag/Delft, the Netherlands (reference: 20250306JKR/wva; approval date: 6 March 2025).

## Declaration of generative AI and AI-assisted technologies

During the preparation of this work the author(s) used Gemini to assist in writing R/codes and ChatGPT in order to improve language and readability of the text. After using this tool/service, the author(s) reviewed and edited the content as needed and take(s) full responsibility for the content of the published article.

## Funding

Julia N. Wolbink is supported by the Dutch Cancer Society (KWF 2023–15744, Implementation of the PORTRET tool in daily clinical practice).

## CRediT authorship contribution statement

**Julia N. Wolbink:** Conceptualization, Formal analysis, Methodology, Writing – original draft. **Leti van Bodegom-Vos:** Conceptualization, Supervision, Writing – review & editing. **Luuk C.A. Ciere:** Formal analysis, Writing – review & editing. **Saskia le Cessie:** Methodology, Writing – review & editing. **Hein Putter:** Formal analysis, Methodology, Writing – review & editing. **Anna Z. de Boer:** Writing – review & editing. **Willeke van der Plas-Krijgsman:** Writing – review & editing. **Frederiek van den Bos:** Writing – review & editing. **Gerrit-Jan Liefers:** Conceptualization, Writing – review & editing. **Johanneke E.A. Portielje:** Conceptualization, Supervision, Writing – review & editing.

## Declaration of competing interest

The authors declare that they have no known competing financial interests or personal relationships that could have appeared to influence the work reported in this paper.

## Figures and Tables

**Table A.1:** **Sensitivity analysis comparing observed and expected 5- and 10-year overall survival under different assumptions:** (1) all women conform the main analysis, (2) all women as smokers, (3) detection occurred through screening instead of symptoms, and (4) chemotherapy, if received, as high-dose, anthracycline- or taxane-based instead of standard-dose, anthracycline-based.

	5 year			10 year		
	Predicted	Observed (s.e.)	Difference (95% CI)	Predicted	Observed (s.e.)	Difference (95% CI)
**Reference**	**81.6**	**74.4 (0.4)**	**7.2 (6.3 to 8)**	**60.3**	**48.2 (0.5)**	**12.1 (11.1 to 13.1)**
*Smoking*	79.5		5.1 (4.3 to 5.9)	56		7.9 (6.9 to 8.8)
*Detection through screening*	83.2		8.8 (8 to 9.6)	62.6		14.4 (13.4 to 15.3)
*If chemotherapy: high dose,*	81.6		7.2 (6.4 to 8)	60.3		12.1 (11.2 to 13.1)
*anthracycline- or taxane-based*						

**Table A.2:** Exploratory analysis showing differences between observed and expected 5-year (A) and 10-year (B) overall survival separately for women aged <75 years and ≥75 years.

A				
	5 year			
	<75 years		≥75 years	
	N(%)	Difference (95% CI)	N(%)	Difference (95% CI)
**N of comorbidities**				
0	862 (17.6%)	0.1 (−1.9 to 2.1)	734 (12%)	1.8 (−1.4 to 5)
1	759 (15.5%)	5.2 (2.6 to 7.8)	971 (15.8%)	7.7 (4.8 to 10.7)
2	515 (10.5%)	5.1 (2 to 8.2)	746 (12.1%)	11.2 (7.8 to 14.7)
3	216 (4.4%)	8 (2.7 to 13.2)	404 (6.6%)	17.2 (12.4 to 22)
4	59 (1.2%)	19.6 (7.8 to 31.3)	145 (2.4%)	23.2 (15 to 31.3)
≥5	32 (0.7%)	34.8 (17.6 to 51.9)	65 (1.1%)	34 (22.2 to 45.7)
Unkown	2456 (50.1%)	2.6 (1.2 to 3.9)	3077 (50.1%)	10.5 (8.8 to 12.2)
**Tumor size (mm)**				
1-10	1228 (25.1%)	1.5 (−0.1 to 3)	506 (8.2%)	2.2 (−1 to 5.4)
11-20	2319 (47.3%)	3.2 (1.9 to 4.6)	2087 (34%)	8 (6.1 to 9.9)
21-30	916 (18.7%)	4.9 (2.4 to 7.4)	2199 (35.8%)	11.6 (9.6 to 13.7)
>30	436 (8.9%)	7.2 (2.9 to 11.5)	1350 (22%)	13.9 (11.2 to 16.5)
**Tumor grade**				
1	1432 (29.2%)	3.4 (1.8 to 4.9)	1344 (21.9%)	9.5 (7.2 to 11.9)
2	2355 (48.1%)	2.8 (1.5 to 4.1)	2969 (48.3%)	9.6 (8 to 11.3)
3	1112 (22.7%)	5.1 (2.6 to 7.6)	1829 (29.8%)	11.3 (9 to 13.6)
**Positive nodes (N)**				
0	3567 (72.8%)	2.3 (1.3 to 3.3)	3696 (60.2%)	9 (7.6 to 10.5)
1-3	1003 (20.5%)	5.6 (3.2 to 8)	1633 (26.6%)	12.4 (10 to 14.7)
≥4	329 (6.7%)	9.5 (4.3 to 14.7)	813 (13.2%)	10.5 (7.1 to 13.9)
**Estrogen receptor status**				
Negative	622 (12.7%)	3.9 (0.4 to 7.3)	1025 (16.7%)	7.7 (4.7 to 10.8)
Positive	4277 (87.3%)	3.4 (2.4 to 4.4)	5117 (83.3%)	10.6 (9.3 to 11.8)
**Progesterone receptor status**				
Negative	1483 (30.3%)	3.3 (1.4 to 5.3)	2128 (34.6%)	9.9 (7.8 to 12)
Positive	3416 (69.7%)	3.5 (2.4 to 4.6)	4014 (65.4%)	10.2 (8.8 to 11.6)
**HER2 receptor status**				
Negative	4477 (91.4%)	3.5 (2.5 to 4.5)	5459 (88.9%)	10.8 (9.5 to 12)
Positive	422 (8.6%)	3.4 (−0.6 to 7.4)	683 (11.1%)	4.9 (1.1 to 8.6)
**Adjuvant endocrine therapy**				
No	2837 (57.9%)	2.8 (1.6 to 4.1)	2747 (44.7%)	9.2 (7.4 to 11)
Yes	2062 (42.1%)	4.3 (2.8 to 5.9)	3395 (55.3%)	10.9 (9.3 to 12.4)
**Adjuvant chemotherapy**				
No	4712 (96.2%)	3.5 (2.5 to 4.4)	6101 (99.3%)	10.2 (9 to 11.4)
Yes	187 (3.8%)	3.8 (−2.6 to 10.3)	41 (0.7%)	−6.1 (−21.2 to 9)
**Adjuvant radiotherapy**				
No	1746 (35.6%)	5.8 (4.1 to 7.6)	3731 (60.7%)	13.8 (12.2 to 15.4)
Yes	3153 (64.4%)	2.2 (1 to 3.3)	2411 (39.3%)	4.4 (2.6 to 6.2)
**Adjuvant trastuzumab**				
No	4846 (98.9%)	3.6 (2.7 to 4.6)	6129 (99.8%)	10.1 (8.9 to 11.3)
Yes	53 (1.1%)	−13.6 (−22.7 to −4.5)	13 (0.2%)	11.1 (−15.4 to 37.5)

B				
	10 year			
	<75 years		≥75 years	
	N(%)	Difference (95% CI)	N(%)	Difference (95% CI)
**N of comorbidities**				
0	862 (17.6%)	0.2 (−2.9 to 3.2)	734 (12%)	4.3 (0.4 to 8.2)
1	759 (15.5%)	10 (6.3 to 13.8)	971 (15.8%)	13.3 (9.9 to 16.8)
2	515 (10.5%)	10.5 (5.8 to 15.2)	746 (12.1%)	16.6 (12.9 to 20.3)
3	216 (4.4%)	17.1 (9.8 to 24.4)	404 (6.6%)	26.6 (21.8 to 31.5)
4	59 (1.2%)	19.1 (5.8 to 32.5)	145 (2.4%)	27 (19.3 to 34.6)
≥5	32 (0.7%)	46.5 (30.2 to 62.8)	65 (1.1%)	36.5 (26.1 to 46.8)
Unkown	2456 (50.1%)	7.3 (5.4 to 9.2)	3077 (50.1%)	16.4 (14.7 to 18)
**Tumor size (mm)**				
1-10	1228 (25.1%)	5.2 (2.7 to 7.8)	506 (8.2%)	8.1 (3.5 to 12.6)
11-20	2319 (47.3%)	7.7 (5.7 to 9.6)	2087 (34%)	16.1 (13.9 to 18.3)
21-30	916 (18.7%)	10.6 (7.3 to 13.9)	2199 (35.8%)	16.6 (14.5 to 18.6)
>30	436 (8.9%)	8.4 (3.5 to 13.3)	1350 (22%)	15.9 (13.6 to 18.1)
**Tumor grade**				
1	1432 (29.2%)	8.6 (6.1 to 11)	1344 (21.9%)	18.5 (15.7 to 21.3)
2	2355 (48.1%)	7 (5 to 8.9)	2969 (48.3%)	16.4 (14.6 to 18.3)
3	1112 (22.7%)	8.1 (5 to 11.1)	1829 (29.8%)	11.9 (9.8 to 14)
**Positive nodes (N)**				
0	3567 (72.8%)	6.5 (5 to 8.1)	3696 (60.2%)	16 (14.3 to 17.7)
1-3	1003 (20.5%)	10.6 (7.5 to 13.8)	1633 (26.6%)	17.1 (14.7 to 19.4)
≥4	329 (6.7%)	11.6 (6.2 to 17.1)	813 (13.2%)	10.5 (7.8 to 13.2)
**Estrogen receptor status**				
Negative	622 (12.7%)	7.1 (3 to 11.1)	1025 (16.7%)	11 (8.3 to 13.7)
Positive	4277 (87.3%)	7.8 (6.3 to 9.2)	5117 (83.3%)	16.5 (15.1 to 17.9)
**Progesterone receptor status**				
Negative	1483 (30.3%)	7.7 (5.1 to 10.3)	2128 (34.6%)	13.2 (11.2 to 15.2)
Positive	3416 (69.7%)	7.7 (6.1 to 9.3)	4014 (65.4%)	16.8 (15.2 to 18.4)
**HER2 receptor status**				
Negative	4477 (91.4%)	8 (6.5 to 9.4)	5459 (88.9%)	16.2 (14.9 to 17.5)
Positive	422 (8.6%)	4.7 (−0.2 to 9.7)	683 (11.1%)	10.4 (6.9 to 13.9)
**Adjuvant endocrine therapy**				
No	2837 (57.9%)	6.2 (4.4 to 7.9)	2747 (44.7%)	14 (12.2 to 15.9)
Yes	2062 (42.1%)	9.9 (7.6 to 12.1)	3395 (55.3%)	16.9 (15.2 to 18.6)
**Adjuvant chemotherapy**				
No	4712 (96.2%)	8 (6.6 to 9.3)	6101 (99.3%)	15.7 (14.4 to 16.9)
Yes	187 (3.8%)	1.6 (−6.4 to 9.5)	41 (0.7%)	2.6 (−12.7 to 17.9)
**Adjuvant radiotherapy**				
No	1746 (35.6%)	11.9 (9.5 to 14.3)	3731 (60.7%)	19.1 (17.5 to 20.6)
Yes	3153 (64.4%)	5.4 (3.7 to 7.1)	2411 (39.3%)	10.2 (8.1 to 12.3)
**Adjuvant trastuzumab**				
No	4846 (98.9%)	7.9 (6.6 to 9.3)	6129 (99.8%)	15.5 (14.3 to 16.8)
Yes	53 (1.1%)	−14.5 (−28.4 to −0.6)	13 (0.2%)	29.2 (NaN to NaN)

**Table A.3:** Exploratory analysis restricted to women without recorded comorbidities comparing age-related differences in observed and expected 5- and 10-year overall survival.

	N(%)	5 year			10 year		
		Predicted	Observed (s.e.)	Difference (95% CI)	Predicted	Observed (s.e.)	Difference (95% CI)
**Age**							
<75	862 (54%)	90	89.9 (1)	0.1 (−1.9 to 2.1)	75.7	75.5 (1.6)	0.2 (−2.9 to 3.2)
75-79	332 (20.8%)	83.4	81.3 (2.1)	2.1 (−2.1 to 6.3)	62.1	59 (2.9)	3.1 (−2.6 to 8.9)
80-84	257 (16.1%)	76	76.7 (2.6)	−0.7 (−5.9 to 4.5)	47.3	42.6 (3.4)	4.7 (−1.9 to 11.3)
≥85	145 (9.1%)	60.6	54.9 (4.1)	5.7 (−2.4 to 13.8)	27.2	20.3 (3.7)	6.9 (−0.4 to 14.2)

## References

[1] Wishart G.C., Azzato E.M., Greenberg D.C., Rashbass J., Kearins O., Lawrence G. (2010). PREDICT: a new UK prognostic model that predicts survival following surgery for invasive breast cancer. Breast Cancer Res.

[2] Derks M.G.M., Bastiaannet E., van de Water W., de Glas N.A., Seynaeve C., Putter H. (2018). Impact of age on breast cancer mortality and competing causes of death at 10 years follow-up in the adjuvant TEAM trial. Eur J Cancer.

[3] Derks M.G.M., van de Velde C.J.H., Giardiello D., Seynaeve C., Putter H., Nortier J.W.R. (2019). Impact of comorbidities and age on cause-specific mortality in postmenopausal patients with breast cancer. Oncologist.

[4] Breast P. (2025). https://breast.v3.predict.cam/tool.

[5] Pharoah P.D.P., Hsiao Y.W., Wishart G.C., Peng P.C. (2025). PREDICT breast v4.0: an update to the PREDICT breast prognostic model. BMC Res Notes.

[6] Vreven L.W.A., Verheul E.M., van Maaren M.C., Doornkamp F., Schipper R.-J., Siesling S. (2026). Comparative validation of PREDICT versions 3.1 and 2.2 for overall survival in the Dutch breast cancer population. Breast.

[7] van der Plas-Krijgsman W.G., Giardiello D., Putter H., Steyerberg E.W., Bastiaannet E., Stiggelbout A.M. (2021). Development and validation of the PORTRET tool to predict recurrence, overall survival, and other-cause mortality in older patients with breast cancer in the Netherlands: a population-based study. Lancet Healthy Longev.

[8] Hsiao Y.-W., Wishart G.C., Pharoah P.D.P., Peng P.-C. (2024). Validation of the PREDICT breast version 3.0 prognostic tool in US breast cancer patients. medRxiv.

[9] Bommelé J., Hipple Walters B., Willemsen M. (2023). Smoking in the Netherlands: key statistics for 2022: statistics on smoking, smoking cessation, and the use of e-cigarettes in the Netherlands. https://www.trimbos.nl/wp-content/uploads/2023/08/AF2091-Smoking-in-the-Netherlands-key-statistics-for-2022.pdf.

[10] Winton Centre for Risk & Evidence Communication at Cambridge University (2025). predictv30r. https://github.com/WintonCentre/predictv30r.

[11] McLernon D.J., Giardiello D., Van Calster B., Wynants L., van Geloven N., van Smeden M. (2023). Assessing performance and clinical usefulness in prediction models with survival outcomes: practical guidance for cox proportional hazards models. Ann Intern Med.

[12] de Boer A.Z., Bastiaannet E., Putter H., Marang-van de Mheen P.J., Siesling S., de Munck L. (2021). Prediction of other-cause mortality in older patients with breast cancer using comorbidity. Cancers (Basel).

[13] de Boer A.Z., van der Hulst H.C., de Glas N.A., Marang-van de Mheen P.J., Siesling S., de Munck L. (2020). Impact of older age and comorbidity on locoregional and distant breast cancer recurrence: a large population-based study. Oncologist.

[14] de Glas N.A., Bastiaannet E., Engels C.C., de Craen A.J.M., Putter H., van de Velde C.J.H. (2016). Validity of the online PREDICT tool in older patients with breast cancer: a population-based study. Br J Cancer.

[15] Adesoye T., Liao K.P., Peterson S., Li L., Zorzi D., Holmes H.M. (2023). Patient-reported outcomes in older breast cancer survivors with and without prior chemotherapy treatment. Cancer Med.

[16] Depboylu B. (2020). Treatment and patient related quality of life issues in elderly and very elderly breast cancer patients. Transl Cancer Res.

[17] de Glas N., Bastiaannet E., de Boer A., Siesling S., Liefers G.J., Portielje J. (2019). Improved survival of older patients with advanced breast cancer due to an increase in systemic treatments: a population-based study. Breast Cancer Res Treat.

[18] HMD. Human Mortality Database [Internet] (2025). https://www.mortality.org.

[19] pengpclab (2025). PREDICTv4.1.1. https://github.com/pengpclab/PREDICTv4.1.1.

